# Patterns and trends in eczema management in UK primary care (2009–2018): A population‐based cohort study

**DOI:** 10.1111/cea.13783

**Published:** 2020-11-23

**Authors:** Simon de Lusignan, Helen Alexander, Conor Broderick, John Dennis, Andrew McGovern, Clarie Feeney, Carsten Flohr

**Affiliations:** ^1^ Nuffield Department of Primary Care health Sciences University of Oxford Oxford UK; ^2^ Royal College of General Practitioners, Research and Surveillance Centre London UK; ^3^ Unit for Population‐Based Dermatology Research St John's Institute of Dermatology, Guy's & St Thomas' NHS Foundation Trust and King's College London London UK; ^4^ Momentum Data Pendragon House St. Albans UK; ^5^ Pfizer Ltd Tadworth UK

**Keywords:** atopic dermatitis, atopic eczema, dermatology, eczema, epidemiology, primary care, treatment

## Abstract

**Background:**

Despite the high disease burden of eczema, a contemporary overview of the patterns and trends in primary care healthcare utilization and treatment is lacking.

**Objective:**

To quantify primary care consultations, specialist referrals, prescribing, and treatment escalation, in children and adults with eczema in England.

**Methods:**

A large primary care research database was used to examine healthcare and treatment utilization in people with active eczema (*n* = 411,931). Management trends and variations by age, sex, socioeconomic status, and ethnicity were described from 2009 to 2018 inclusive.

**Results:**

Primary care consultation rates increased from 87.8 (95% confidence interval [95% CI] 87.3–88.3) to 112.0 (95% CI 111.5–112.6) per 100 person‐years over 2009 to 2018. Specialist referral rates also increased from 3.8 (95% CI 3.7–3.9) to 5.0 (95% CI 4.9–5.1) per 100 person‐years over the same period. Consultation rates were highest in infants. Specialist referrals were greatest in the over 50s and lowest in people of lower socioeconomic status, despite a higher rate of primary care consultations. There were small changes in prescribing over time; emollients increased (prescribed to 48.5% of people with active eczema in 2009 compared to 51.4% in 2018) and topical corticosteroids decreased (57.3%–52.0%). Prescribing disparities were observed, including less prescribing of potent and very potent topical corticosteroids in non‐white ethnicities and people of lower socioeconomic status. Treatment escalation was more common with increasing age and in children of non‐white ethnicity.

**Conclusion and clinical relevance:**

The management of eczema varies by sociodemographic status in England, with lower rates of specialist referral in people from more‐deprived backgrounds. There are different patterns of healthcare utilization, treatment, and treatment escalation in people of non‐white ethnicity and of more‐deprived backgrounds.

## 1 INTRODUCTION

Eczema (syn. “atopic dermatitis,” “atopic eczema”) is the most common inflammatory skin condition worldwide in children,^1^ and persistence of chronic disease into adulthood is common.^2^, ^3^ Eczema can be extremely disabling and has a significant psychological impact in both children and adults.^4^, ^5^, ^6^

In the UK, the burden of eczema management falls on primary care. Attendance rates are high, with 96% of children with eczema reported to have had a primary care attendance within the preceding year.^7^ Whilst contemporary UK population‐based estimates of eczema disease severity are lacking, a 1998 cross‐sectional analysis in a UK general practice setting estimated 16% of eczema in children aged 1–5 to be moderate or severe.^7^ Recent studies of children with eczema actively recruited from UK primary care suggest this figure may be even higher.^8^, ^9^ US population‐based studies have reported 7% of children and 11% of adults with eczema have severe disease.^10^, ^11^ Whilst those with more severe disease are more likely to be referred for specialist care, the majority of these cases are managed without secondary care referral.^7^ As a result, the principal costs for eczema are those of primary care attendances and prescribing.^12^

Standard topical eczema care includes regular emollient application. Escalation to topical corticosteroids (TCS), or, as an alternative, topical calcineurin inhibitors (TCI), is common for maintenance and flare management,^13^, ^14^ and topical antimicrobials can be used to address secondary skin infections, or pruritis.^14^ Antihistamines are also commonly used to treat pruritus associated with eczema, although evidence for their effectiveness is limited.^15^ In more severe disease, systemic immuno‐modulatory treatment may be required.^16^, ^17^ Recently, the first biologic therapy for eczema, dupilumab, was approved, and this can now be prescribed for adults and adolescents with moderate or severe eczema who have not responded or have contraindications to conventional systemic therapies.^18^

Despite the high disease burden and multiple treatments available, a contemporary overview of UK primary care healthcare and treatment utilization patterns and trends in children and adults with eczema is lacking. We set out to describe healthcare utilization in people with eczema across the lifespan, including primary care attendances, specialist referrals, prescribing and, as a surrogate marker of moderate and severe eczema, treatment escalation patterns.^19^

## 2 METHODS

### 2.1 Data source and setting

We used the Oxford‐Royal College of General Practitioners (RCGP) Research and Surveillance Centre (RSC) network database. The RCGP RSC comprises the pseudonymized primary care records of all individuals registered with a large network of general practices, providing a broadly representative sample of the English primary care population.^20^ Over the entire study period, the RCGP RSC database contained data from 3.85 million people registered with 293 general practitioner (GP) practices across England.

RCGP RSC primary care records include information on demographics, clinical diagnoses, laboratory tests, prescriptions, and care processes (eg patient referrals). Data are captured using the Read coding system (a thesaurus of clinical terms).^21^ Key strengths of the English primary care system include: it is a registration‐based system (each patient registers with a single GP), has been computerized since the 1990s, laboratory results are electronically uploaded, and, since 2004, a pay‐for‐performance scheme has resulted in high‐quality chronic disease clinical data entry.^22^, ^23^ Additionally, RCGP RSC practices have practice visits and receive feedback via a dashboard to improve data quality.^24^

All children and adults registered with an RCGP RSC contributing practice between 1 January 2009 and 1 January 2019 were eligible for inclusion in this study. Individuals required at least 1 year of follow‐up in RCGP RSC, unless under 1‐year old. The full protocol for the study was pre‐specified and has been previously published.^25^

### 2.2 Eczema definitions

People with eczema were identified using Read diagnostic codes and prescription records, applying a validated algorithm recently developed in a random sample of children and adults in UK primary care, and previously applied in several UK primary care studies.^19^, ^26^, ^27^ The positive predictive value of this algorithm for a physician‐confirmed diagnosis of eczema is 90% (95% confidence interval (CI) 80%–91%) in children and 82% (95% CI 73%–89%) in adults.^27^

Active eczema was defined, as in a recent UK primary care study,^19^ as two eczema records (either diagnoses or treatment) appearing within any 1‐year period. Active eczema was then assumed to last for 1 year, unless another eczema record appeared, in which case its duration was prolonged for a further 1 year.^19^ We utilized this approach but used the first of two codes (rather than the latter) within 1 year to signify the onset of active eczema, as this has been shown to have good agreement to physician‐confirmed onset,^27^ and has been used elsewhere.^26^

Eczema treatments are also prescribed for other conditions, and the indication for treatment is not readily available in primary care data. We therefore excluded people with potential confounding comorbidities from our eczema cohort. We excluded people who had other skin conditions (psoriasis, contact dermatitis, photodermatitis, and ichthyosis) as these are managed with similar topical treatments to eczema.^27^ In addition, we excluded people with inflammatory bowel disease (IBD), rheumatoid arthritis, and a history of organ transplantation, as these are commonly managed with treatments also used in eczema (eg methotrexate and azathioprine); topical treatments for dermatological conditions and oral immuno‐modulating drugs for the other conditions listed. IBD and rheumatoid arthritis were identified using validated approaches.^28^, ^29^, ^30^ Organ transplantation was identified using a Read code list generated in accordance with publicly available guidance.^21^, ^31^

### 2.3 Definition of sociodemographic factors

Ethnicity was categorized in accordance with the major UK census categories: white, Asian, black African/Caribbean, mixed, other, and not recorded. Deprivation was defined using the official national measure of socioeconomic status, the Index of Multiple Deprivation (IMD).^32^ Scores, based on postcode, were stratified by deprivation quintile according to the national distribution.

### 2.4 Primary care visits and specialist referrals

The reason for primary care attendances is not always coded in the primary care record. To define primary care attendances specifically for eczema, we matched primary care appointment dates to the dates of prescriptions issued for eczema treatment (as defined below). A primary care attendance for eczema was defined as either a visit where an eczema diagnosis code was recorded or a prescription for eczema treatment was issued. Specialist referrals were identified by the presence of a Read code for referral to either a dermatologist, a GP with a specialty interest in dermatology, or a dermatology specialist nurse.

### 2.5 Treatment and treatment escalation

We extracted prescription records for therapy classes commonly used to manage eczema in the UK: emollients and soap substitutes (combined into a single therapy class for the purposes of analysis), TCS, TCI, systemic immuno‐modulatory therapy (ciclosporin, azathioprine, methotrexate, mycophenolate, and oral corticosteroids), oral antihistamines, and topical antimicrobial treatments.

In line with published work, we used treatment escalation as a surrogate marker to define moderate and severe eczema.^19^ We analysed three elements of treatment escalation: (1) time to a prescription of a second potent topical corticosteroid treatment within 1 year or a first topical calcineurin inhibitor (moderate eczema); (2) time to a systemic immuno‐modulatory therapy or a dermatology referral (severe eczema); and (3) time to a first systemic immuno‐modulatory therapy (as an important component of [2]).

### 2.6 Statistical analyses

#### 2.6.1 Definition of the prevalent cohort

Prevalent individuals were those fulfilling the diagnostic criteria for active eczema at the 31 December of each calendar year of the study.

#### 2.6.2 Primary care visits and specialist referrals

Within the prevalent cohort, we described annual rates of primary care attendances and specialist referrals for eczema. In the prevalent cohort in 2018, we calculated stratified rates of attendances and referrals by age category, sex, ethnicity, and deprivation quintile.

#### 2.6.3 Treatment patterns

We described the patterns of eczema treatment in the prevalent cohort. Results were stratified by the same sociodemographic factors and calendar year. The proportion of the prevalent cohort receiving each medication class was calculated as the number of the prevalent cohort receiving at least one prescription for a particular medication class during a year divided by the total number in the prevalent cohort for that year. As a sensitivity analysis, we repeated the analysis for antihistamine prescriptions in the subset of people with active eczema without a clinical diagnosis of allergic rhinitis, as antihistamines are commonly prescribed for this condition.

#### 2.6.4 Definition of the incident cohort

To evaluate treatment escalation, a subset of people with incident eczema were identified as those diagnosed with new‐onset eczema over the study period. Patients with an eczema diagnosis recorded in their primary care record prior to the study period were excluded from this incident cohort.

#### 2.6.5 Treatment escalation

In the incident cohort, we examined each of the three treatment escalation outcomes using time to event analysis, separately analysing children and adults. First, we compared the cumulative incidence of each outcome by age at diagnosis category (age groups: 0–1, 2–11, 12–17, 18–49, and 50+) using the Kaplan‐Meier estimator. Second, we examined the non‐linear effect of continuous age (modelled as a restricted cubic spline with 3 knots) using multivariable Cox proportional hazards models, with adjustment for sex, ethnicity and deprivation quintile. The impact of age category was also evaluated using adjusted Cox regression. Follow‐up began on the date of diagnosis and extended to the earliest of the study end‐date (1 January 2019), the date of patient transfer from an included practice, date of death, or the date an individual developed an outcome of interest.

Statistical analyses were performed using R version 3.4.1 (R Core Team, Vienna, Austria, 2017).

### 2.7 Ethics approval

Study approval was granted by the Research Committee of the RCGP RSC. The study did not meet the requirements for formal ethics board review as defined using the NHS Health Research Authority research decision tool (http://www.hra‐decisiontools.org.uk/research/).

The study was conducted following RECORD (REporting of studies Conducted using Observational Routinely‐collected Data) guidelines.^33^

## 3 RESULTS

**FIGURE 1:**
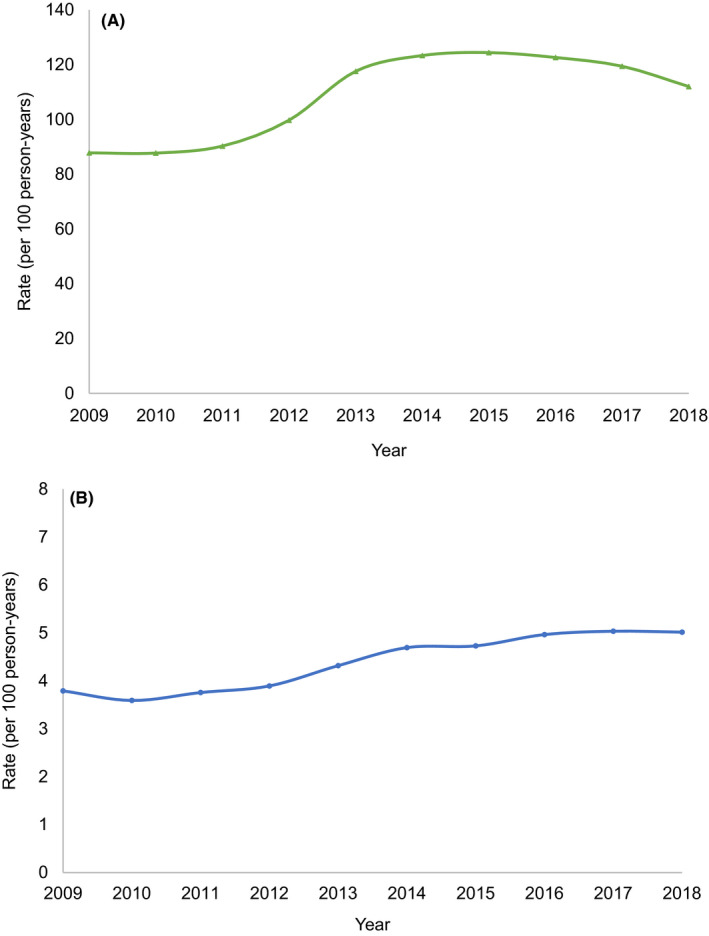
The annual rates of eczema primary care consultations and specialist referrals for eczema between 2009 and 2018 (*n* = 411,931). A, Primary care consultations. B, Specialist referrals for eczema

411,931 individuals in the RCGP RCS database met the definition of active eczema for at least 1 year over 2009 to 2018 inclusive (Flowchart S1). Annual rates of primary care consultations for eczema increased over the study period, from 87.8 (95% CI 87.3; 88.3) consultations per 100 person‐years in 2009 to 112.0 (95% CI 111.5; 112.6) per 100 person‐years in 2018 (Figure 1A). Specialist dermatology referral rates increased from 3.8 (95% CI 3.7; 3.9) per 100 person‐years in 2009 to 5.0 (95% CI 4.9; 5.1) per 100 person‐years in 2018 (Figure 1B).

### 3.1 Rates of eczema consultations and specialist referrals differ by age and sociodemographic factors

In people with active eczema in 2018 (*n* = 148,166), rates of eczema consultations were markedly higher in children under 2 than in other age groups, but rates of specialist referrals were highest in adults aged 50 or over (Table 1). Rates of eczema consultations were similar by sex, but were higher in all non‐white ethnicity categories. There was little difference in the rates of specialist referrals by sex or ethnicity. Primary care eczema consultation rates were highest for those in the most deprived quintile. However, rates of specialist referrals were highest in the least deprived quintile. Whilst there was no difference in rates of specialist referral between rural and urban areas, GP consultation rates were higher in urban settings.

**TABLE 1:** Primary care attendance rates and specialist referral rates for eczema by sociodemographic factors, in people with active eczema in 2018 (*n* = 148,166)

	Number of individuals with eczema	Eczema consultation rates (per 100 person‐years)	Specialist referral rates (per 100 person‐years)
Overall	148,166	112.0 (111.5, 112.6)	5.0 (4.9, 5.1)
Age group			
<2	7230	213.3 (211.7, 215.2)	5.8 (5.2, 6.4)
2–11	34,074	95.6 (94.6, 96.7)	2.4 (2.3, 2.6)
12–17	12,476	84.3 (82.7, 86.0)	3.8 (3.4, 4.1)
18–49	37,995	106.2 (105.2, 107.3)	5.8 (5.5, 6.0)
≥50	56,391	119.8 (118.8, 120.7)	6.3 (6.1, 6.5)
Sex			
Female	81,357	113.8 (113.0, 114.5)	5.1 (4.9, 5.3)
Male	66,809	109.9 (109.1, 110.7	4.9 (4.7, 5.1)
IMD quintile^a^			
1 (most deprived)	26,454	131.2 (129.8, 132.6)	4.5 (4.2, 4.8)
2	25,520	118.6 (117.3, 120.0)	4.8 (4.6, 5.1)
3	26,445	110.0 (108.7, 111.2)	5.0 (4.8, 5.3)
4	31,426	105.3 (104.1, 106.4)	5.1 (4.9, 5.4)
5 (least deprived)	36,467	101.2 (100.1, 102.2)	5.4 (5.1, 5.6)
Ethnicity^b^			
White	87,717	108.6 (107.9, 109.3)	5.0 (4.8, 5.2)
Asian	14,980	138.3 (136.4, 140.2)	5.3 (4.9, 5.7)
Black	5799	116.7 (113.9, 119.6)	4.4 (3.9, 5.0)
Mixed	2808	116.0 (112.0, 120.1)	4.6 (3.8, 5.5)
Other	1234	124.5 (118.3, 131.0)	6.2 (4.9, 7.8)
Rural‐urban classification^c^			
Urban	117,466	103.1 (101.9, 1.04.3)	5.1 (4.8, 5.3)
Rural	29,005	114.3 (113.7, 115.0)	5.0 (4.9, 5.1)

Abbreviation: IMD = index of multiple deprivation.

^a^ IMD data were not available for *n* = 1854.

^b^ Ethnicity data were not available for *n* = 35,628.

^c^ Rural‐Urban classification was not available for *n* = 1695.

### 3.2 Prescribing for eczema has changed little but varies markedly by age and sociodemographic characteristics

**FIGURE 2:**
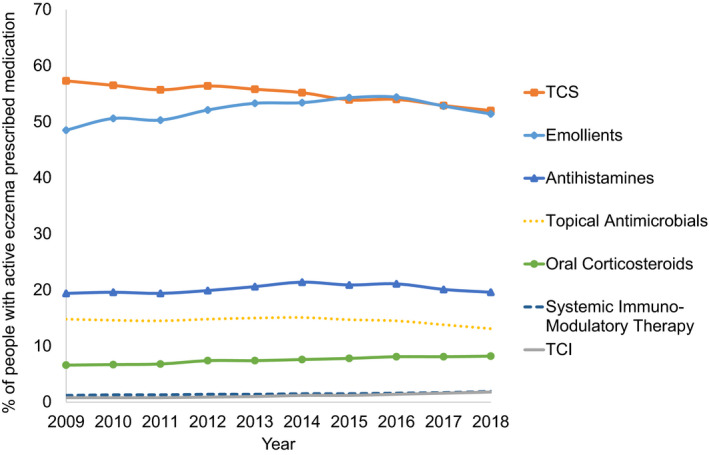
The annual prevalence of prescribing for eczema by therapy class between 2009 and 2018 (*n* = 411,931)

Over 2009–2018 (*n* = 411,931 with active eczema), the most common therapy classes prescribed were emollients and TCS (Figure 2), and there was a slight increase in prescribing of emollients (prescribed to 51.4% of people in 2018 compared to 48.5% in 2009) and a decrease in prescribing of TCS (52.0% 2018, 57.3% in 2009) over the 10‐year study period. There were also increases in prescribing of TCI therapy (1.8% in 2018, 0.8% in 2009), systemic immuno‐modulatory therapy (1.9% in 2018, 1.2% in 2009), and oral corticosteroids (8.2% in 2018, 6.6% in 2009), but little change in antihistamine prescribing (19.6% in 2018, 19.4% in 2009) and a reduction in prescribing of topical antimicrobials (13.1% in 2018, 14.8% in 2009).

**FIGURE 3:**
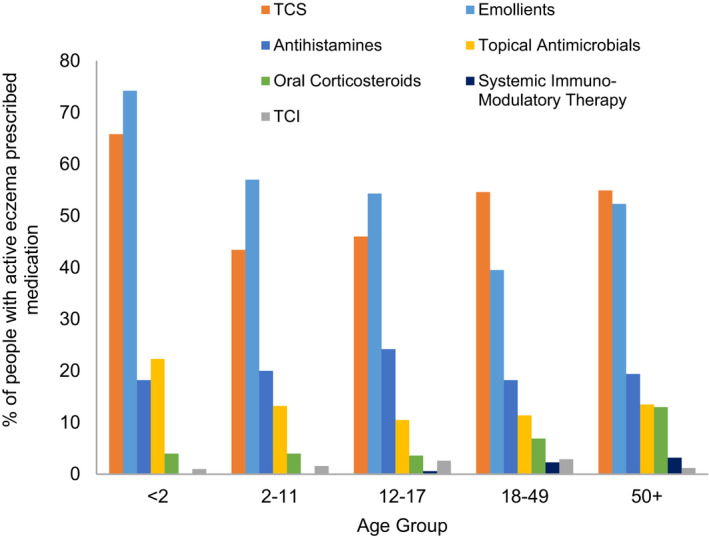
The prevalence of prescribing for eczema by age group and by therapy class for people with active eczema in 2018 (*n* = 148,166)

In people with active eczema in 2018 (*n* = 148,166), emollients were the most commonly prescribed treatment in children, whilst TCS were the most common treatment in adults (Table 2 and Figure 3). Mild TCS were commonly prescribed in children under 2, whilst use of very potent TCS was mainly restricted to adults. Antihistamines and oral corticosteroids were the most common systemic therapies prescribed, with oral corticosteroids more frequently prescribed in adults than children.

**TABLE 2:** Prescribing of therapy by sociodemographic factors for people with active eczema in 2018 (*n* = 148,166)

	*N*	Therapy class									
		Emollients and soap substitutes	Mild TCS	Moderate TCS	Potent TCS	Very potent TCS	TCI	Topical antimicrobials	Antihistamines	Systemic immuno‐modulatory therapy	Oral corticosteroids
		*N* (%)	*N* (%)	*N* (%)	*N* (%)	*N* (%)	*N* (%)	*N* (%)	*N* (%)	*N* (%)	*N* (%)
Overall	148,166	76 095 (51.4%)	40 478 (27.3%)	19 575 (13.2%)	34 997 (23.6%)	6478 (4.4%)	2683 (1.8%)	19 378 (13.1%)	29 041 (19.6)	2785 (1.9)	12 087 (8.2)
Age group											
<2	7230	5368 (74.2)	4384 (60.6)	1135 (15.7)	635 (8.8)	18 (0.2)	73 (1.0)	1614 (22.3)	1315 (18.2)	0 (0)	291 (4.0)
2–11	34,074	19 431 (57.0)	11 256 (33.0)	3967 (11.6)	3431 (10.1)	176 (0.5)	530 (1.6)	4508 (13.2)	6811 (20.0)	31 (0.1)	1353 (4.0)
12–17	12,476	6777 (54.3)	3425 (27.5)	1686 (13.5)	2260 (18.1)	208 (1.7)	326 (2.6)	1307 (10.5)	3022 (24.2)	73 (0.6)	455 (3.6)
18–49	37,995	15 016 (39.5)	8529 (22.4)	5520 (14.5)	11 568 (30.4)	2127 (5.6)	1096 (2.9)	4333 (11.4)	6932 (18.2)	860 (2.3)	2629 (6.9)
≥50	56,391	29 503 (52.3)	12 884 (22.8)	7267 (12.9)	17 103 (30.3)	3949 (7)	658 (1.2)	7616 (13.5)	10 961 (19.4)	1821 (3.2)	7359 (13)
Sex											
Male	81,357	41 139 (50.6)	22 237 (27.3)	10 692 (13.1)	18 658 (22.9)	3832 (4.7)	1489 (1.8)	10 960 (13.5)	16 394 (20.2)	1704 (2.1)	6905 (8.5)
Female	66,809	34 956 (52.3)	18 241 (27.3)	8883 (13.3)	16 339 (24.5)	2646 (4.0)	1194 (1.8)	8418 (12.6)	12 647 (18.9)	1081 (1.6)	5182 (7.8)
IMD quintile^a^											
1 (most deprived)	26,454	14 785 (55.9)	7761 (29.3)	3425 (12.9)	5834 (22.1)	1029 (3.9)	499 (1.9)	3765 (14.2)	6413 (24.2)	404 (1.5)	2270 (8.6)
2	25,520	13 784 (54)	7182 (28.1)	3380 (13.2)	5832 (22.9)	1048 (4.1)	469 (1.8)	3393 (13.3)	5480 (21.5)	378 (1.5)	2002 (7.8)
3	26,445	13 273 (50.2)	6977 (26.4)	3540 (13.4)	6410 (24.2)	1197 (4.5)	454 (1.7)	3440 (13)	4944 (18.7)	541 (2)	2212 (8.4)
4	31,426	15 538 (49.4)	8350 (26.6)	4039 (12.9)	7691 (24.5)	1472 (4.7)	532 (1.7)	3986 (12.7)	5563 (17.7)	684 (2.2)	2581 (8.2)
5 (least deprived)	36,467	17 738 (48.6)	9648 (26.5)	4921 (13.5)	8761 (24)	1659 (4.5)	690 (1.9)	4554 (12.5)	6259 (17.2)	745 (2)	2853 (7.8)
Ethnicity^b^											
White	87,717	42 490 (48.4)	22 791 (26)	11 437 (13)	21 859 (24.9)	4227 (4.8)	1353 (1.5)	11 496 (13.1)	16 214 (18.5)	1913 (2.2)	8311 (9.5)
Asian	14,980	8900 (59.4)	4188 (28)	2133 (14.2)	3502 (23.4)	671 (4.5)	513 (3.4)	2005 (13.4)	4064 (27.1)	211 (1.4)	975 (6.5)
Black	5799	3659 (63.1)	1409 (24.3)	683 (11.8)	1154 (19.9)	196 (3.4)	122 (2.1)	660 (11.4)	1486 (25.6)	50 (0.9)	241 (4.2)
Mixed	2808	1660 (59.1)	836 (29.8)	385 (13.7)	561 (20)	92 (3.3)	73 (2.6)	344 (12.3)	637 (22.7)	30 (1.1)	129 (4.6)
Other	1234	638 (51.7)	340 (27.6)	166 (13.5)	265 (21.5)	46 (3.7)	34 (2.8)	192 (15.6)	301 (24.4)	12 (1)	56 (4.5)

Abbreviation: IMD, index of multiple deprivation.

^a^ IMD data were not available for *n* = 1854.

^b^ Ethnicity data were not available for *n* = 35,628.

Fewer people of non‐white ethnicities and people from the two most deprived IMD quintiles were prescribed potent and very potent TCS (Table 2). People of non‐white ethnicities were also more likely to be prescribed emollients, TCI or antihistamines, and less likely to be prescribed oral corticosteroids. People from less deprived IMD quintiles were less likely to be prescribed emollients or antihistamines. Antihistamine prescribing was lower in people with active eczema without a clinical diagnosis of allergic rhinitis (overall prescribing prevalence 15.1% versus 19.6% in all people with active eczema, but prescribing time trends and differences in antihistamine prescribing by sociodemographic factors in this subgroup were consistent with the whole active eczema population (Figure S1, Table S1).

### 3.3 Treatment escalation

**FIGURE 4:**
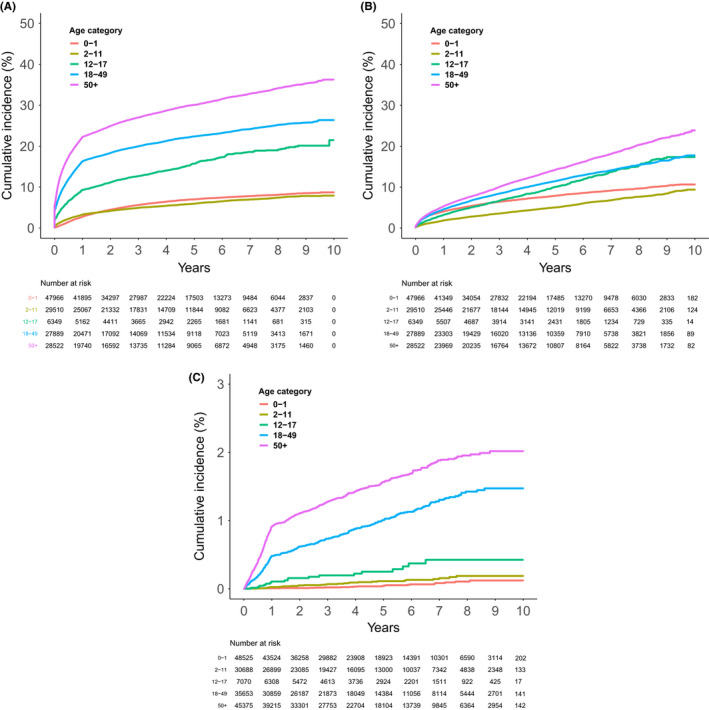
Cumulative incidence of treatment escalation by age at diagnosis category in people with incident eczema over 2009–2018. A, Cumulative incidence of the prescription of a second potent topical corticosteroid treatment within 1 year or a first topical calcineurin inhibitor (TCI) (moderate eczema) in 140,236 people without moderate or severe eczema at diagnosis. B, Cumulative incidence of the first of systemic immuno‐modulatory therapy or a dermatology referral (severe eczema) in 140,236 people without moderate or severe eczema at diagnosis. C, Cumulative incidence of initiation of systemic immuno‐modulatory therapy (Ciclosporin, Azathioprine, Methotrexate & Mycophenolate mofetil) in 167,311 people with incident eczema

167,311 people with incident eczema were included in the analysis of treatment escalation. Incidence of moderate and severe eczema was higher in adolescents than younger children (Figure 4A,B, Table 3A), with both peaking at age 18 when age was analysed as a continuous measure (Figure S2). In adults, incidence of moderate and severe eczema increased with a higher age at diagnosis, although the difference in incidence by age was more marked in moderate than severe eczema (Figure 4A,B, Table 3B, Figure S2). Initiation of systemic immuno‐modulatory therapy was low overall, was higher in adults than children (Figure 4C), and in adults was highest in those aged 50 to 68, before declining at older ages (Figure S2). 10‐year absolute risks of progressing to each end point are reported, by age category, in Table S2.

**TABLE 3:** Hazard ratios (95% confidence intervals) for time to moderate eczema and severe eczema in eligible populations with incident eczema over 2009–2018, by sociodemographic characteristics. Individuals with each outcome at baseline excluded from analysis of each outcome

	Moderate eczema	Severe eczema
**(A) Children**	**(*n* = 84,819)**	**(*n* = 84,819)**
Sex		
Female	1.00 (ref)	1.00 (ref)
Male	1.10 (1.04, 1.16)	1.01 (0.96, 1.07)
Age category		
<2	1.00 (ref)	1.00 (ref)
11‐Feb	0.92 (0.87, 0.98)	0.65 (0.61, 0.69)
17‐Dec	2.85 (2.78, 3.07)	1.31 (1.20, 1.43)
IMD quintile^a^		
1 (most deprived)	1.00 (ref)	1.00 (ref)
2	1.02 (0.95, 1.11)	1.14 (1.05, 1.25)
3	0.87 (0.79, 0.94)	1.15 (1.05, 1.26)
4	0.92 (0.85, 1.01)	1.13 (1.03, 1.23)
5 (least deprived)	0.90 (0.83, 0.98)	1.22 (1.12, 1.32)
Ethnicity^a^		
White	1.00 (ref)	1.00 (ref)
Asian	1.71 (1.58, 1.86)	1.82 (1.67, 1.98)
Black	1.54 (1.37, 1.73)	1.49 (1.31, 1.69)
Mixed	1.41 (1.21, 1.65)	1.59 (1.36, 1.85)
Other	1.57 (1.25, 1.97)	1.47 (1.15, 1.89)
**(B) Adults**	**(*n* = 55,417)**	**(*n* = 55,417)**
Sex		
Female	1.00 (ref)	1.00 (ref)
Male	1.31 (1.27, 1.36)	0.96 (0.91, 1.01)
Age category		
18–49	1.00 (ref)	1.00 (ref)
50+	1.40 (1.35, 1.45)	1.24 (1.18, 1.31)
IMD quintile^b^		
1 (most deprived)	1.00 (ref)	1.00 (ref)
2	1.00 (0.95, 1.07)	1.05 (0.96, 1.14)
3	1.04 (0.99, 1.11)	1.11 (1.01, 1.21)
4	0.99 (0.94, 1.05)	1.18 (1.09, 1.29)
5 (least deprived)	0.97 (0.92, 1.03)	1.26 (1.16, 1.37)
Ethnicity^b^		
White	1.00 (ref)	1.00 (ref)
Asian	1.12 (1.06, 1.19)	0.97 (0.88, 1.07)
Black	0.89 (0.79, 0.99)	0.86 (0.73, 1.02)
Mixed	1.05 (0.88, 1.25)	1.16 (0.91, 1.49)
Other	0.88 (0.72, 1.07)	0.89 (0.66, 1.19)

^a^ Hazard ratios for IMD not recorded 1.02 (95% CI 0.81, 1.29) for moderate eczema and 1.02 (0.78, 1.32) for severe eczema; Hazard ratios for Ethnicity not recorded category 1.08 (1.01, 1.15) for moderate eczema and 1.29 (1.21, 1.37) for severe eczema.

^b^ Hazard ratios for IMD not recorded 1.03 (0.87, 1.21) for moderate eczema, 1.26 (1.00, 1.59) for severe eczema; Hazard ratios for Ethnicity not recorded category 0.97 (0.93, 1.01) for moderate eczema, 0.96 (0.90, 1.02) for severe eczema.

In children, there was a greater risk of treatment escalation in people of non‐white ethnicity. In adults, only in people of Asian ethnicity was there evidence of a greater risk of progression to moderate but not severe eczema (Table 3). Males were more likely to progress to moderate eczema than females (Table 3). Children and adults from the least deprived category were more likely to progress to severe eczema. Conversely, children from the least deprived category were less likely to progress to moderate eczema. For adults, there was no evidence of a difference in progression to moderate eczema by deprivation quintile. As initiation of systemic immuno‐modulatory therapy was uncommon, stratification by sociodemographic characteristics was not possible for this end point.

## 4 DISCUSSION

Our study provides important insight into the primary care management and disease course of eczema across the lifespan. Both primary care consultation and specialist referral rates for eczema increased over the last decade, and we observed substantial differences in consultation rates by age, ethnicity and deprivation category. Notable findings include that, although primary care consultation rates for eczema were highest in the people of lower socioeconomic status, people of higher socioeconomic status were more likely to have a specialist referral for eczema. We also observed higher primary care consultation rates in non‐white ethnicities, with the highest rates seen in people from an Asian background. Whilst over the study period there was little change in overall treatment patterns, treatment escalation varied by socioeconomic status and ethnicity, and in particular was more common in children of non‐white ethnicity.

### 4.1 Strengths and limitations

Strengths of this study include the use of contemporary data from a large population‐representative primary care cohort with high‐quality ethnicity and deprivation data. Accurate prescribing data are ensured through automatic entry when prescriptions are generated. A limitation of our study is the likelihood that some patients included in the incident eczema cohort did not have true new‐onset eczema, as they had a historical onset of eczema prior to our study period that was not captured in the primary care record. Although diagnosis dates can be retrospectively coded in UK primary care, this may not always be done correctly. This limitation will be most applicable to older patients, for example if they had childhood eczema at a time before the introduction of electronic health records which has not been subsequently retrospectively coded. This limitation is common to all studies using primary care databases to define incident disease cohorts. A further limitation is that prescriptions issued in secondary care are not available in UK electronic health record data, and as a result, our study will have systematically undercaptured prescribing of systemic immuno‐modulatory therapies. We are also likely to have underestimated specialist dermatology referral rates in this study, as it is likely that some dermatology referrals will have been coded as unspecified referrals without mention of clinical speciality and were therefore not included in our estimates. In addition, we found that phototherapy data captured in RCGP RCS were not complete enough to be used for analysis, and these data were therefore not included in our study.

Our definitions of active, moderate and severe eczema are concordant with recently published work exploring outcomes for people with eczema,^19^ but the accuracy of this approach to classify eczema has not been validated against physician‐assessed disease severity. Similarly, although the algorithm used to identify eczema has been demonstrated to have high positive predictive value, the negative predictive value, sensitivity and specificity has not been assessed.^27^ It is likely that some patients self‐managing with very mild eczema will have been missed using the algorithm, and so the healthcare utilization measures in this study should be interpreted as estimates applicable for patients in contact with primary care physicians to manage their eczema. Future studies incorporating our findings must acknowledge this limitation and be cautious if they seek to generalize to the population of all patients with eczema, including the very mildest, self‐managed forms. This could be especially relevant for health economics analysis, as to neglect to do so could exaggerate the healthcare resources required by patients with eczema. The definitions for treatment escalation are in‐line with a previous systematic review which proposed that an eczema flare should be defined as an episode requiring treatment escalation or seeking additional medical advice.^34^ Nonetheless, as the reason underlying prescribing decisions is not captured, our data cannot elucidate why treatment was escalated. Performance of the diagnostic algorithm has also not been assessed as yet in sociodemographic subgroups, and a potential explanation of the observed differences in treatment escalation is variation in algorithm performance across such different population groups. A more comprehensive evaluation of the performance of the eczema diagnostic algorithm, as well as evaluation of definitions of eczema severity, progression and escalation in primary care, would be useful areas for further work.

### 4.2 Context of previous work

The increase in primary and secondary care consultations concur with a large retrospective analysis of overall GP clinical workload in the UK, which found that annual consultation rates increased by 10.5% between 2007 and 2014.^35^ A rising elderly population and a doubling of telephone consultations were among possible explanations.

To our knowledge, there have been no large‐scale studies of overall GP clinical workload from 2014 onwards. We observed a plateau in consultation rates. This may have resulted from an emphasis placed on self‐management of chronic conditions in recent years, leading to a reduced requirement for doctor‐patient interactions.^36^ Written action plans have been demonstrated to be an effective means of patient education in eczema, improving understanding of both the condition and its treatment.^37^

We found specialist referrals for eczema were highest in the over 50s age group. A qualitative study of GP's experiences of eczema stated that whilst most GPs feel confident in diagnosing uncomplicated eczema, many report uncertainty in diagnosing and managing more complex cases, particularly where potent steroids are required.^38^ This may explain the increase in referral rates for eczema among the elderly, who are often resistant to standard treatments.^39^

Our observation of disparities in referral to secondary care by socioeconomic status fits with the inverse care law (that those who most need medical care are least likely to receive it^40^) and has been observed in studies examining GP referral patterns for other conditions. McBride et al observed people of higher socioeconomic status had higher rates of specialist referral for hip pain and dyspepsia, postulating that this might reflect higher workloads for practitioners in socially disadvantaged communities.^41^ In addition, patients from more‐deprived areas have a greater burden of multi‐morbidity and present to GP consultations with more problems to discuss^42^; potentially diverting the focus of the encounter away from eczema.

The relatively stable prescribing patterns we observed for eczema are in contrast to the major changes seen in prescribing for other conditions in recent years, such as type 2 diabetes.^43^ This likely reflects the fact that UK National Institute for Health and Care Excellence (NICE) treatment guidelines for eczema,^14^ in contrast to those for type 2 diabetes,^44^ have not been updated since 2007. A potential explanation for the decline in prescribing of emollients in recent years is recently updated UK prescribing recommendations, which for dry skin conditions (although not eczema itself) encourage over the counter purchases of emollients instead of the provision of routine prescriptions.^45^

Differences in prescribing patterns by age found in this study are broadly in‐line with treatment guidelines and previous studies. US population‐based studies have estimated that 7% of children and 11% of adults with eczema have severe disease.^10^, ^11^ Our finding of 8%–10% of under 12s prescribed potent TCS is therefore likely in‐line with national guidelines, which recommend potent TCS are to be used in moderate to severe eczema.^14^, ^46^

Our study shows important differences in prescribing by socioeconomic status, with a greater rate of topical antimicrobial, antihistamine and oral steroid prescribing in more‐deprived groups, and lower rates of potent TCS and systemic immuno‐modulatory treatment use. This may be related in part to higher levels of specialty referral with increasing socioeconomic status, as seen in our study and others.^47^ GPs are very unlikely or unable to prescribe oral immuno‐suppressive agents without secondary care initiation and shared care agreements.

Differences in prescribing by ethnicity were also observed; emollient use was higher in those from non‐white ethnicities, whereas oral steroid and other systemic therapies were more frequently prescribed to those from a white background. Important nuances in the visual appearance of eczema in non‐white skin may lead to under‐estimation of its severity and hence under treatment.^48^, ^49^ Higher rates of antihistamine prescriptions in black and Asian populations fit with previous studies showing higher rates of pruritus, scratching and subsequent lichenification in these groups.^49^

## 5 CLINICAL IMPLICATIONS

Our study highlights important health disparities in the management of eczema in England that warrant further study. Of particular note are the lower rates of both referrals to secondary care and prescriptions of topical and systemic immuno‐modulatory treatments in those from more‐deprived backgrounds, and the greater risk of progression to moderate and severe eczema in children of non‐white ethnicity. It is unlikely that individual GPs are aware of the disparities in referral, and feedback of practice‐level data to GPs via dashboards and observatories may be able to raise awareness of such disparities.^50^

Although prescribing trends were consistent over the study period, it will be important to monitor prescribing behaviour in primary and secondary care over the coming years due to likely changes in prescribing guidelines and the availability of new systemic treatments.

## CONFLICT OF INTEREST

S. de Lusignan is Director of the Royal College of General Practitioners Research and Surveillance Centre as part of his academic post; he has also received funding for projects from Eli Lilly, AstraZeneca, GSK, Seqirus, and Takeda, all through his universities and none related to this study. C. Feeney is an employee of Pfizer. J. Dennis and A. McGovern are employees of Momentum Data who were paid consultants to Pfizer in connection with the development of this manuscript. C. Flohr is chief investigator of the UK National Institute for Health Research–funded TREAT (ISRCTN15837754) and SOFTER (ClinicalTrials.gov: NCT03270566) trials and the UK‐Irish Atopic Eczema Systemic Therapy Register (A‐STAR; ISRCTN11210918) and is a principal investigator in the European Union Horizon 2020–funded BIOMAP Consortium (http://www.biomap‐imi.eu/). His department has also received funding from Sanofi‐Genzyme. All other authors have no competing interests to declare.

## AUTHOR CONTRIBUTION

The study concept and design were developed by C. Flohr, C. Feeney, H. Alexander, A. McGovern, J. Dennis, and S. de Lusignan. The study was performed and written under the direction of S. de Lusignan, C. Feeney, H. Alexander, C. Broderick, and C. Flohr. J. Dennis and A. McGovern conducted and are responsible for the data analysis. All authors critically reviewed the manuscript. S. de Lusignan had full access to all the data in the study and takes responsibility for the integrity of the data and the accuracy of the data analysis.

## Supporting information

Appendix S1

## Data Availability

The RCGP RSC data set is held securely at University of Oxford and the University of Surrey and can be accessed by bone fide researchers. Approval is on a project‐by‐project basis (www.rcgp.org.uk/rsc). Ethical approval by an NHS Research Ethics Committee may be needed before any data release/other appropriate approval. Researchers wishing to directly analyse the patient‐level pseudonymized data will be required to complete information governance training and work on the data from university secure servers. Patient‐level data cannot be taken out of the secure network.
