# Supplementary material for: Patterns and trends in eczema management in UK primary care (2009–2018): A population‐based cohort study
Source: Clin Exp Allergy. 2020 Nov 23;51(3):483–94. doi: 10.1111/cea.13783 (PMC7984383; doi:10.1111/cea.13783)

**Supplementary material for:** Patterns and trends in eczema management in UK primary care (2009-2018): a population-based cohort study

**Authors:** S. de Lusignan, H. Alexander, C. Broderick, J. Dennis, A. McGovern, C. Feeney, C. Flohr

**Supplementary Flowchart 1:** Flowcharts for construction of study population

**
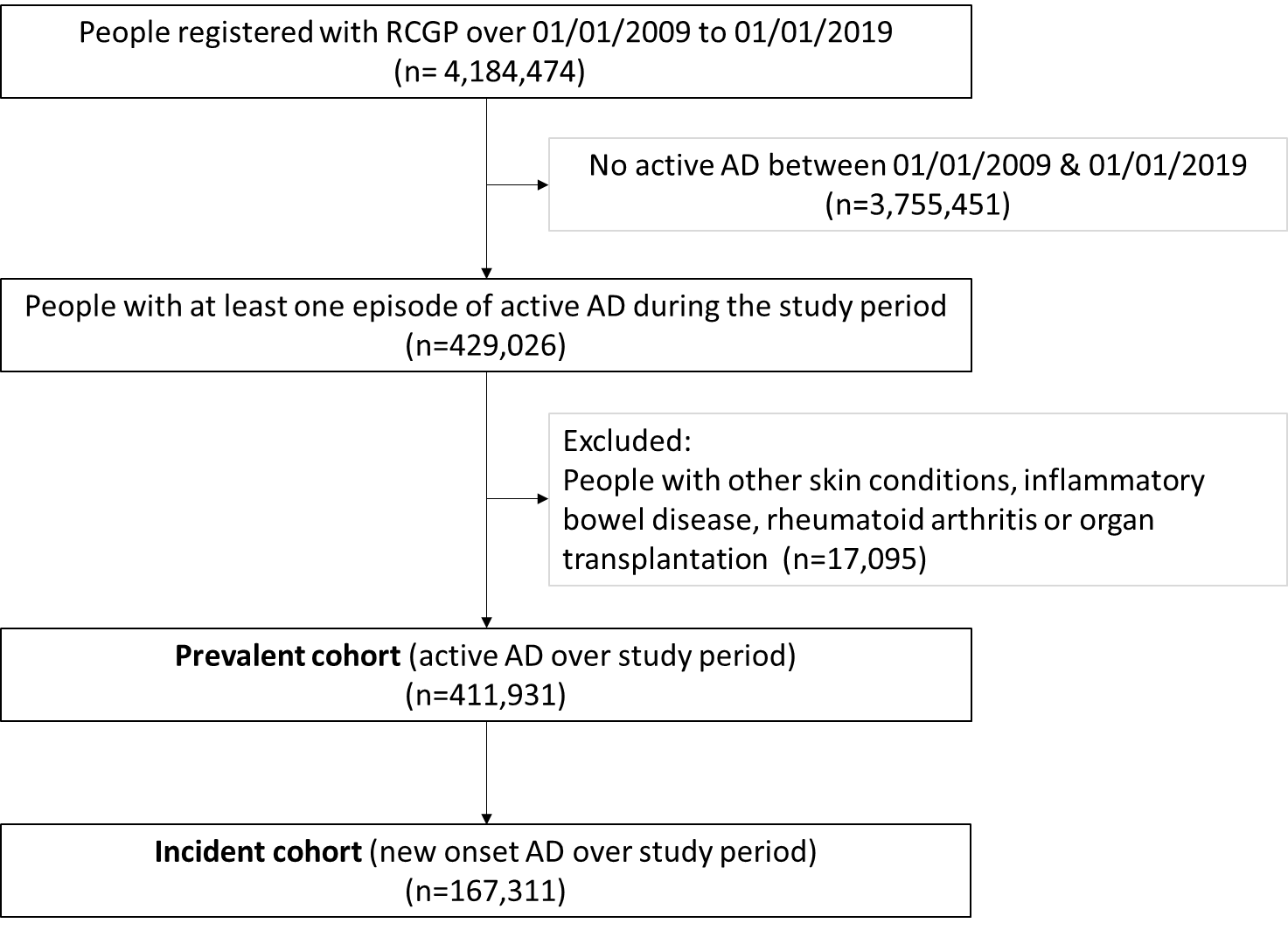
**

**Supplementary Figure 1:** The annual prevalence of antihistamine prescribing for eczema between 2009-2018, in all patients with eczema (n=411,931) and the subset of patients with active eczema and no clinical diagnosis of allergic rhinitis (n=332,275)

**
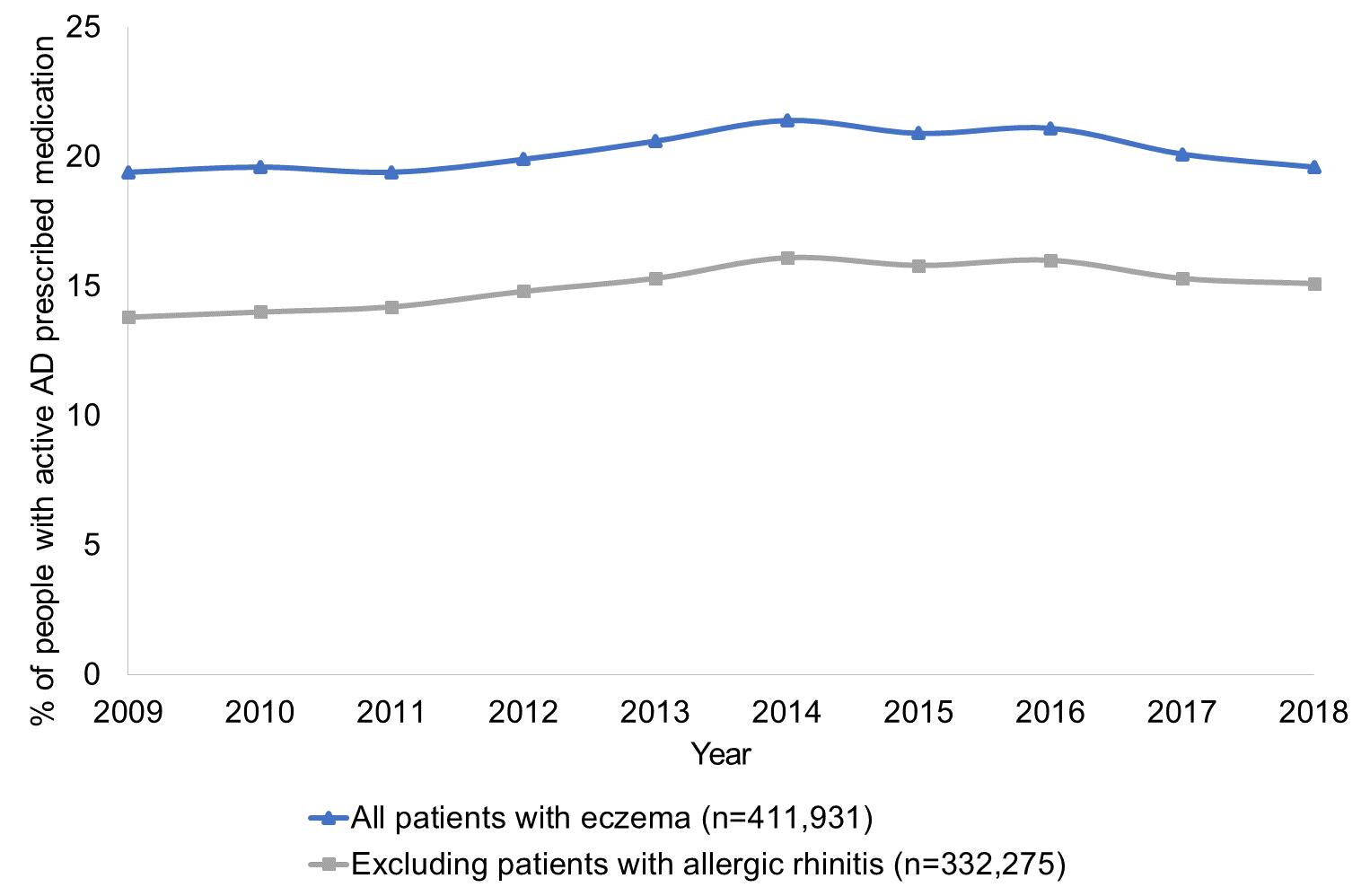
**

**Supplementary Table 1:** Prescribing of antihistamine therapy by sociodemographic factors for people with active eczema in 2018, in all patients with eczema (n=148,166) and the subset of patients with active eczema and no clinical diagnosis of allergic rhinitis (n=117,375)

|  | All patients with active eczema  (n=148,166) | | Patients with active eczema and no clinical diagnosis of allergic rhinitis (n=117,375) | | |
| --- | --- | --- | --- | --- | --- |
|  | **N (%)** | **N prescribed antihistamine (%)** | | **N (%)** | **N prescribed antihistamine (%)** |
| Overall | **148,166** | **29041**  **(19.6)** | | **117,375** | **17697 (15.1)** |
| Age group |  |  | |  |  |
| <2 | 7,230 | 1315 (18.2) | | 7,140 | 1263 (17.7) |
| 2-11 | 34,074 | 6811 (20.0) | | 30,520 | 4921 (16.1) |
| 12-17 | 12,476 | 3022 (24.2) | | 9,026 | 1371 (15.2) |
| 18-49 | 37,995 | 6932 (18.2) | | 25,967 | 3,188 (12.3) |
| ≥50 | 56,391 | 10961 (19.4) | | 44,722 | 6,954 (15.5) |
| Sex |  |  | |  |  |
| Male | 81,357 | 16394 (20.2) | | 63,953 | 9,913 (15.5) |
| Female | 66,809 | 12647 (18.9) | | 53,422 | 7,784 (14.6) |
| IMD quintile* |  |  | |  |  |
| 1 (most deprived) | 26,454 | 6413 (24.2) | | 20,643 | 4,036 (19.6) |
| 2 | 25,520 | 5480 (21.5) | | 20,206 | 3,397 (16.8) |
| 3 | 26,445 | 4944 (18.7) | | 21.053 | 2,967 (14.1) |
| 4 | 31,426 | 5563 (17.7) | | 25,057 | 3,376 (13.5) |
| 5 (least deprived) | 36,467 | 6259 (17.2) | | 28,915 | 3,667 (12.7) |
| Ethnicity† |  |  | |  |  |
| White | 87,717 | 16214 (18.5) | | 69,610 | 9,904 (14.2) |
| Asian | 14,980 | 4064 (27.1) | | 10,870 | 2,288 (21.0) |
| Black | 5,799 | 1486 (25.6) | | 4,133 | 779 (18.8) |
| Mixed | 2,808 | 637 (22.7) | | 2,172 | 366 (16.9) |
| Other | 1,234 | 301 (24.4) | | 962 | 181 (18.8) |

**Supplementary Table 2:** 10 year absolute risk of the three treatment escalation endpoints in people with incident AD, by age category. Values are percentages (95% confidence intervals)

|  | Moderate AD  (n=140,236)* | Severe AD  (n=140,236)* | First systemic therapy  (n=167,311)** |
| --- | --- | --- | --- |
| Age category |  |  |  |
| <2 | 8.7 (8.3, 9.1) | 10.6 (10.2, 11.1) | 0.12 (0.06, 0.18) |
| 2-11 | 7.9 (7.4, 8.4) | 9.4 (8.7, 10.1) | 0.19 (0.11, 0.27) |
| 12-17 | 21.5 (18.4, 24.4) | 17.4 (15.6, 19.1) | 0.42 (0.21, 0.64) |
| 18-49 | 26.4 (25.6, 27.2) | 17.8 (16.9, 18.7) | 1.47 (1.28, 1.66) |
| 50+ | 36.3 (35.3, 37.2) | 23.9 (22.6, 25.1) | 2.02 (1.83, 2.20) |

*Individuals without moderate or severe AD at diagnosis

**All individuals with incident AD


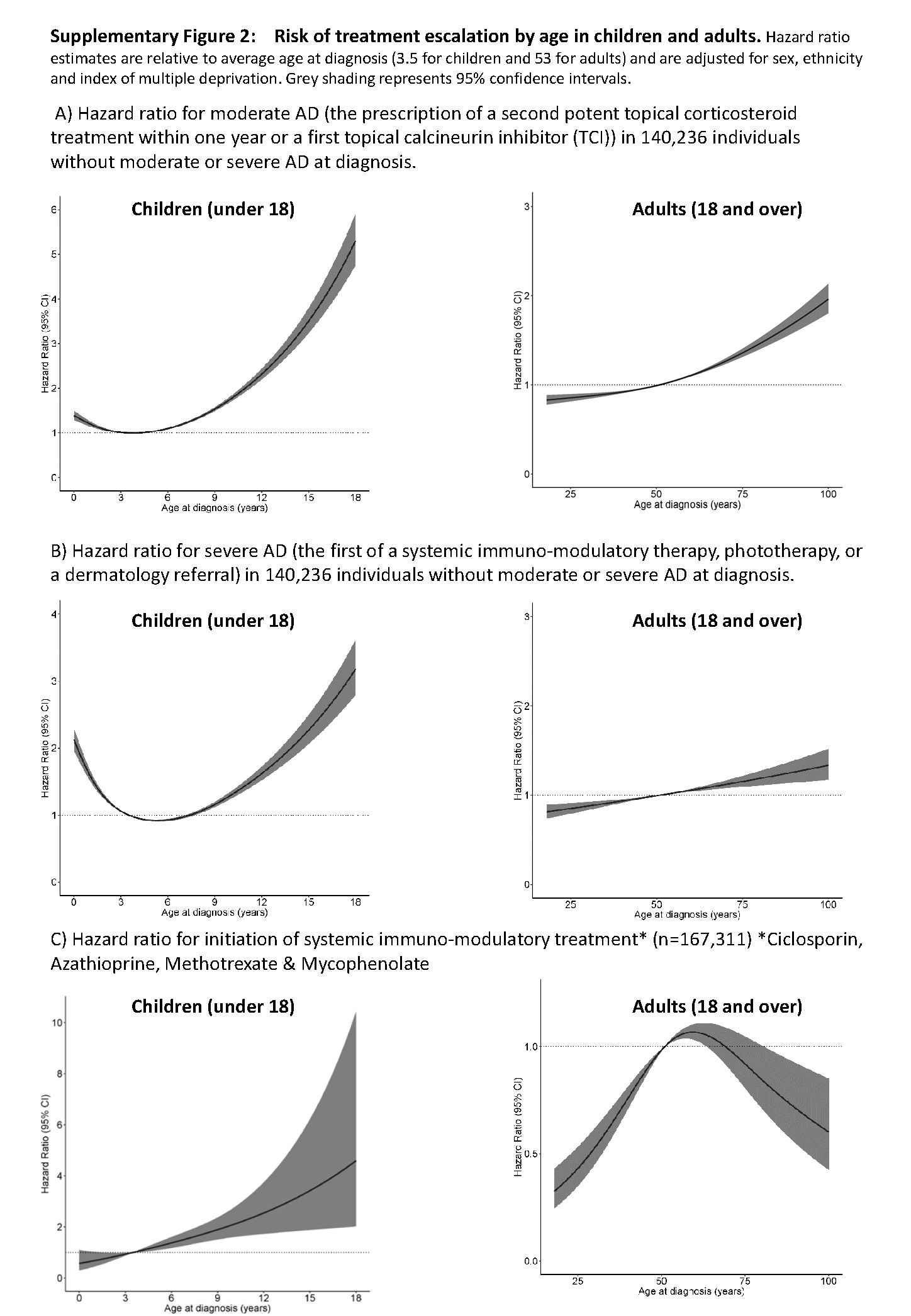

Supplement: Supplementary file 1 — Appendix S1 [file CEA-51-483-s001.docx]
